# An integrative approach for rapid authentication of different parts of *Camellia petelotii* (Merr.) Sealy: combining ATR-FTIR spectroscopy with conventional chemometric analysis and a convolutional neural network

**DOI:** 10.3389/fchem.2026.1830739

**Published:** 2026-08-19

**Authors:** Chen Jingying, Zhao Yunqing, Zhang Wujun, Huang Yingzhen, Wan Yin Tew, Jingsong Liu, Liyun Ouyang, Qiyue Qiu, Chen Ying, Tiem Leong Yoon, Mun Fei Yam

**Affiliations:** 1 Crop Research Institute, Fujian Academy of Agricultural Sciences, Fuzhou, Fujian, China; 2 School of Pharmaceutical Sciences, Universiti Sains Malaysia, Minden, Pulau Pinang, Malaysia; 3 College of Pharmacy, Fujian University of Traditional Chinese Medicine, Fuzhou, Fujian, China; 4 School of Physics, Universiti Sains Malaysia, Minden, Pulau Pinang, Malaysia; 5 Faculty of Business and Communications, INTI International University, Nilai, Negeri Sembilan, Malaysia

**Keywords:** chemometric analysis, convolutional neural network, data augmentation, machine learning, spectroscopic analysis

## Abstract

**Introduction:**

The flower of *Camellia petelotii* (Merr.) Sealy is highly valued in Traditional Chinese Medicine, making it susceptible to adulteration with its leaves or other lower-cost adulterants. This study aims to develop a robust authentication approach to distinguish different plant parts and identify adulterated mixtures using ATR-FTIR spectroscopy integrated with chemometrics and artificial intelligence.

**Methods:**

The flowers, leaves, seeds, and mixed samples of *C. petelotii* were subjected to ATR-FTIR spectroscopy. The spectral data of pure plant parts were analyzed with Principal Component Analysis (PCA) and Orthogonal Partial Least Squares Discriminant Analysis (OPLS-DA). In the machine learning phase, a Convolutional Neural Network (CNN) model was trained on spectra of pure plant parts and laboratory-prepared mixed samples. The Synthetic Minority Oversampling Technique (SMOTE) was employed to address class imbalance and sample scarcity. Model robustness was validated via Repeated Random Subsampling Validation (RRSV).

**Results:**

ATR-FTIR analysis clearly differentiated the leaves from other plant parts, revealing distinct spectral characteristics. The PCA and OPLS-DA effectively classified the three distinct plant parts with high accuracy, sensitivity, and specificity scores exceeding 95%. The OPLS-DA model achieved high internal validity (R^2^X, R^2^Y, and Q^2^Y ≥ 0.738). In the machine learning workflow, the baseline CNN models suffered from minority-class collapse. Implementing SMOTE effectively resolved this issue. In multi-class configurations, the SMOTE-trained models showed high predictive precision and achieved high F1-scores for Seed (0.846) and Flower (0.931) classes, but moderate performance on Mix (0.593) and Leaf (0.657) classes. The binary classification model resolved the ambiguity, increasing the average F1-scores of the Mix and Leaf classes to 0.674 and 0.932, respectively. When validated against non-augmented data across both multi-class and binary-class configurations, SMOTE-trained models showed high stability for pure plant parts but remained sensitive to the Mix class across both multi-class (F1-score: 0.361) and binary-class (F1-score: 0.249) configurations.

**Conclusion:**

While AI-driven data augmentation mitigates sample-size constraints, classification performance remains heavily influenced by spectral characteristics. The binary classification architecture offered distinct advantages when analyzing samples with heterogeneous spectral complexity. This integrated approach may serve as a reference for quality control and authentication of botanical products.

## 1 Introduction

*Camellia petelotii* (Merr.) Sealy (heterotypic synonym: *Camellia nitidissima* C.W. Chi) is commonly known as “the queen of camellias” or “dreaming camellia” due to its striking golden-yellow flowers (Min et al., 2007). It is a highly esteemed species widely distributed in Guangxi Zhuang Autonomous Region of China. It is documented in the Compendium of Materia Medica (Ben Cao Gang Mu) and has been traditionally used to treat various conditions, including nephritis, hepatitis, jaundice, urinary tract infections, dysentery, hypertension, diarrhea, liver cirrhosis, sores, and irregular menstruation. Numerous studies have shown that *C. petelotii* has a wide range of pharmacological properties, including anticancer, antioxidant, hypolipidemic, antidiabetic, antiallergic, antibacterial, and anxiolytic activities. These therapeutic effects are mainly attributed to its various bioactive components, such as saponins, flavonoids, polyphenols, vitamins, amino acids, and polysaccharides (An et al., 2020; He et al., 2017; Truong et al., 2024). These properties have positioned it as a candidate for the production of traditional medicines and tea (Huang et al., 2022). Studies have shown that *Camellia petelotii* contains a higher number of phenolic compounds compared to other species in the same genus, including *Camellia sinensis*. This contributes to its therapeutic efficacy and potential for pharmacological application (Lin et al., 2013; Wang et al., 2017).

The growing scientific interest in the therapeutic applications of golden *Camellia* necessitates consistent taxonomic nomenclature to ensure accurate cross-referencing of findings reported across global research. Nomenclatural consistency is important in botanical pharmacology, as historical taxonomic revisions often result in the coexistence of multiple scientific names, leading to considerable ambiguity in the literature. Taxonomic overlap has long existed between *C. petelotii* (Merrill) Sealy (*C. petelotii*) and *Camellia nitidissima* C.W. Chi (*C. nitidissima*). A phylogenomic study stated that *C. nitidissima* is a conspecific of *C. petelotii* (Liu et al., 2025). This relationship is reflected in taxonomic authorities such as Flora of China and major taxonomic databases, including Royal Botanic Gardens, Kew, and World Flora Online, which recognize *C. nitidissima* as a heterotypic synonym of *C. petelotii* (Min et al., 2007; Zhou et al., 2023). Consequently, the accepted binomial *C. petelotii* is used to avoid taxonomic ambiguity.

While standardizing taxonomic nomenclature addresses discrepancies in the literature, a critical challenge remains in the marketplace. The high therapeutic value of *C. petelotii* has made it a valuable ingredient in traditional medicine and health products. The commercial price of *C. petelotii* flowers ranges from 2,000 to 3,000 Chinese renminbi (RMB) per kilogram (kg), compared with 200–400 RMB for leaves, increasing the risk of adulteration. Unethical sellers may adulterate the flowers by mixing them with leaves or other related species in commercial products to increase their profit margins. Chromatographic approaches are widely employed in laboratory research to study chemical composition but are usually time-consuming and involve intricate sample preparation procedures. Consequently, it can be challenging to differentiate between leaves and flowers that have similar phytochemical profiles (Peerapattana et al., 2015; Yang et al., 2013). Ensuring the purity and authenticity of market-available *C. petelotii* products is crucial for preserving their therapeutic efficacy and consumer trust.

Fourier transform infrared spectroscopy (FTIR) is an analytical technique that captures the molecular composition of organic substances by measuring the infrared radiation-induced intramolecular vibrations. FTIR spectroscopy can detect a diverse range of phytochemicals in a single scan (Moros et al., 2010; Tew et al., 2022). Conventional potassium bromide (KBr)-FTIR requires sample destructive pellet preparation, enabling complete infrared transmission and analysis of macroscopic internal properties. In contrast, the non-destructive attenuated total reflectance (ATR)-FTIR technique focuses on the outermost layer of the sample, providing complementary surface-concentrated spectral information that enriches the overall chemical profile while allowing direct preservation of the samples (Moros et al., 2010; Movasaghi et al., 2008; Sharma et al., 2020). The chemical fingerprinting of the seed, leaves, and flowers of *C. petelotii* (formerly *C. nitidissima*) has been explored using conventional KBr-FTIR in previous studies (Tew et al., 2022). However, the baseline chemical fingerprint obtained from KBr-FTIR alone is insufficient to map the complex whole-plant chemical profile. The internal macroscopic transmission-based information lacks the surface-level structural resolution needed to trace subtle variations in surface-concentrated compounds.

Despite the potential of ATR-FTIR spectroscopy for botanical chemical profiling, its primary limitations in robust classification stem from the inherent complexity of plant phytochemical profiles and biological variability. Raw mid-infrared spectra often show overlapping vibrational bands, where the dominant absorption peaks from macromolecules mask the low-abundance secondary metabolites that differ across distinct samples (Eissa et al., 2024). Additionally, ATR-FTIR is highly susceptible to environmental and instrumental interferences, which lead to baseline drift, light scattering, and environmental moisture noise that disrupt the absolute absorbance values and compromise model reproducibility (Liu et al., 2023). These conditions introduce high-dimensional multicollinearity that affects the performance of traditional chemometrics when the spectral variables greatly outstrip the available sample size (Calvo-Gomez et al., 2022). Traditional chemometrics often fail to resolve overlapping, non-linear signals. Furthermore, standard chemometric approaches fail to handle uneven data distributions, leading the classification model to be biased toward the dominant sample group when sample availability is naturally limited.

To address these limitations, the present study introduces a novel, integrated analytical strategy characterized by a sequential workflow. Rather than relying on simple multivariate visualization, this study progresses systematically from initial raw ATR-FTIR spectral differentiation to conventional chemometric cluster analysis, and subsequently to a deep learning Convolutional Neural Network (CNN) architecture executed after targeted synthetic data augmentation. The distinct novelty of this combined workflow lies in its dual-targeted resolution. The Synthetic Minority Oversampling Technique (SMOTE) was applied to eliminate sample size imbalances across different plant parts, while a one-dimensional CNN (1D-CNN) framework was engineered to capture complex, non-linear spectral features without manual feature selection (Acquarelli et al., 2017; Elreedy et al., 2023; Mohanty et al., 2025; Yang et al., 2019). Crucially, this study utilizes a newly acquired, completely independent dataset with no sample or spectral overlap with any previously published research. By pairing the surface-sensitive variations of ATR-FTIR with the advanced pattern-recognition capabilities of a 1D-CNN framework, this study establishes a highly scalable, automated botanical authentication model capable of resolving complex spectral ambiguities that traditional chemometric models fail to resolve.

## 2 Methodology

### 2.1 Sample collection

*C. petelotii* samples were collected between August 2017 and August 2018, from Fangchenggang in Guangxi Autonomous Region and Zhangping, Yongfu, and Shanghang in Fujian Province. The complete sample pool comprised 80 flowers, 78 leaves, and 8 seed specimens. All samples were authenticated by Professor Chen Jingying from the Crop Research Institute, Fujian Academy of Agricultural Sciences, and deposited in the Herbarium of the Crop Research Institute (voucher number: H2018061). All specimens were dried in an oven at 45 °C for 48 h prior to pulverization to a 200-mesh powder. Subsequently, the powdered samples were stored at 4 °C. The specimens were heated at 45 °C for an additional 6 h before ATR-FTIR analysis (Chen et al., 2025). This was done to ensure optimal contact between the powder and the ATR crystal during analysis.

### 2.2 Acquisition of ATR-FTIR spectra

An appropriate amount of powdered sample was meticulously deposited onto the sample-loading area to ensure complete coverage of the area. A constant force was applied to ensure intimate contact between the sample and the surface. The ATR-FTIR spectra were obtained from 4000 cm^-1^ to 400 cm^-1^ using 32 co-added scans to improve the signal-to-noise ratio. A spectral resolution of 4 cm^-1^ and a sampling interval of 1 cm^-1^ were set to optimize the peak definition. All spectra were baseline-corrected to remove background shifts. Savitzky-Golay polynomial fitting (13-point smoothing) was used to suppress high-frequency noise while preserving the underlying chemical information. Spectral intensities were normalized to eliminate intensity fluctuations caused by inconsistent sample-to-crystal contact pressure, thereby standardizing the dataset for downstream chemometric and machine-learning analysis (Chen et al., 2025; Ng et al., 2018).

### 2.3 Chemometric analysis

During the first stage, principal component analysis (PCA) was used to examine the variance in the FTIR spectral characteristics of different parts of *C. petelotii*. After performing unsupervised Principal Component Analysis (PCA-X), class-specific PCA (PCA-class) and Orthogonal Partial Least Squares Discriminant Analysis (OPLS-DA) were used for discrimination analysis. The samples were divided into two sets, calibration and validation, using a random selection process. The calibration (training) set consisted of 60% of the spectra from different plant parts, while the validation (testing) set included the remaining 40% of each plant part. The specificity, accuracy, and sensitivity of PCA-class and OPLS-DA were evaluated for their ability to differentiate the validation set (Chen et al., 2023; Chen et al., 2025; Tew et al., 2022).

In the OPLS-DA analysis, several model performance parameters were calculated. The R^2^Y represents the amount of variation in the calibration set Y explained by the model, whereas Q^2^Y represents the variation in the calibration set Y that is predicted by the model via cross-validation. In addition, the model accuracy, sensitivity, specificity, the root mean-squared error of estimation (RMSEE), root mean-squared error of cross-validation (RMSECV), and root mean-squared error of prediction (RMSEP) were calculated. The seven-fold cross-validation technique (CV = 7) was applied to assess the model’s robustness and reliability. In addition, an internal validation was conducted using a permutation test with 200 preset permutations. The performance of the calibration model was determined by calculating sensitivity, specificity, and accuracy. The OPLS-DA, PCA, and PCA-class models were created using SIMCA 14.1 (Umetrics, Sweden) (Chen et al., 2023; Chen et al., 2025; Tew et al., 2022). The accuracy, sensitivity, and specificity were calculated as follows:

$$Accuracy=\frac{TN+TP}{Number\;of\;all\;samples}$$

$$Specificity=\frac{TN}{TN+FP}$$

$$Sensitivity/Recall=\frac{TP}{TP+FN}$$

$$Precision=\frac{TP}{TP+FP}$$

where TP = true positive prediction; TN = true negative prediction; FP = false positive prediction; and FN = false negative prediction.

### 2.4 Convolutional neural network model

A Convolutional Neural Network (CNN) was utilized to analyze the ATR-FTIR spectra and perform classification. In the present study, 1-D convolutional layers were employed to process the 1-D FTIR spectral fingerprints. The CNN architecture was composed of two convolutional layers, two max-pooling layers, and two dense activation layers. Each convolutional layer consisted of trainable filters (kernels) that performed convolution operations along the spectral axis, generating activation maps that highlighted the key spectral features. The Rectified Linear Unit (ReLU) activation function was utilized to introduce non-linearity into the convolutional layer outputs, allowing the network to capture complex spectral relationships. Max-pooling operations were then adopted to reduce the dimensionality of the feature maps, thereby decreasing the number of parameters and computational workload (Chen et al., 2025).

Early stopping and batch normalization were incorporated into the training process to prevent overfitting. After feature extraction, a flattening layer was introduced to convert the convolution-generated 2-D arrays into a 1-D vector, making the data compatible with the dense layer. A final softmax layer was added to the model to normalize the model output into a probability distribution across the predefined classes (Chen et al., 2025).

### 2.5 Machine-learning classification using ATR-FTIR spectral fingerprints

#### 2.5.1 The raw data

For machine learning analysis, preprocessed spectra from 166 pure *C. petelotii* samples, along with spectra from an additional 12 laboratory-prepared mixed samples (Table 1), were used. The mixed samples were composite samples prepared by physically homogenizing the flower, leaf, and seed sample powders at mass ratios of 1:1 for binary mixtures and 1:1:1 for ternary mixtures (w/w). These heterogeneous mixtures were formulated using unique, non-redundant individual specimens drawn directly from the authenticated pure samples to simulate complex adulteration scenarios. Although this class does not correspond to a pure component, it is treated as an independent target category in machine learning classification. The compositional imbalance within the raw dataset reflects the true ecological realities, specifically the biological scarcity of seasonal reproductive tissues, such as seeds, and the intentional creation of mixed samples to capture heterogeneous phytochemical profiles. Preserving the intrinsic asymmetry of the raw dataset provides a high-fidelity, real-world evaluation of the model’s true classification capabilities.

**TABLE 1:** Breakdown of the *C. petelotii* samples used in machine learning classification.

Class	Raw sample count
Flower	80
Leaf	78
Seed	8
Mix	12
Total	178

#### 2.5.2 Random partition and data augmentation

To address the class imbalance issue and limited sample availability, the Synthetic Minority Oversampling Technique (SMOTE) was applied to generate synthetic data and increase the sample size (Elreedy et al., 2023; Mohanty et al., 2025). The original real samples for each class were randomly divided into two independent subsets: the training set (Group A) and the evaluation set (Group B), ensuring approximately equal class representation and no overlap. Before generating synthetic data, the exact raw compositions of these subsets were preserved as non-augmented baseline datasets. SMOTE augmentation was then performed on these subsets to generate two corresponding synthetic cohorts: SMOTE-Group A and SMOTE-Group B, with each class oversampled to 500 samples ( $N_{os}=500$ ) (Chen et al., 2025). The oversampling process for each group was conducted independently to avoid data leakage and ensure complete segregation between the training and evaluation datasets. For the seed class, due to the limited sample size and spectral information, an average spectrum was generated by averaging spectral coefficients across the eight genuine seed samples at each wavenumber. This process ensured at least 4 independent seed spectra in each group, meeting the minimum requirement for the SMOTE approach.

#### 2.5.3 Training and evaluation of the CNN-based classifier

To establish a performance baseline, baseline CNN models were initially trained and validated on real, non-augmented Group A, then evaluated against real, non-augmented Group B data. For the augmentation workflow, the SMOTE-Group A was used as the training set for the CNN classifier. Throughout the training phase, stratified 4-fold cross-validation (n_splits = 4) was employed as an intermediate evaluation strategy to optimize model generalization and decrease the risk of overfitting (Chen et al., 2025; Szeghalmy and Fazekas, 2023). The data were equally partitioned into four distinct folds. In each iteration, the model was trained on three folds and validated on the remaining fold (k−1) . The process was repeated four times, generating four discrete models with their own set of training and validation histories.

The CNN models trained with SMOTE-Group A (SMOTE-trained) were evaluated in two distinct phases to ensure clear separation between synthetic performance and true biological predictive ability. To assess classification efficacy on synthetic data, the SMOTE-trained models were evaluated against augmented data (SMOTE-Group B). To evaluate the model’s generalization, the generated SMOTE-trained models were evaluated against real, non-augmented held-out Group B samples. This separation ensures that performance on augmented samples is clearly separated from the model’s true generalization on real-world data.

The model’s performance was evaluated using F1-score derived from the confusion matrix. This metric was computed with the following formulas:

$$F1‐Score=2\times \frac{Precision\times Recall}{Precision+Recall\;}$$

where TP = true positive prediction; FP = false positive prediction. The metric is calculated per class.

#### 2.5.4 Repeated Random Subsampling Validation and classification strategy

Repeated Random Subsampling Validation (RRSV) was conducted to evaluate the robustness and performance consistency of the CNN classifier (Chen et al., 2025). In this procedure, the entire modelling process was repeated seven times (n_run = 7), each with newly randomized training–testing partitions of the dataset. Both training and testing subsets were regenerated and oversampled independently for each run to ensure complete independence of the data across all repetitions. At the end of the RRSV, 28 CNN models were evaluated. The consistency of class-wise performance metrics across runs was used to validate the CNN model’s robustness. Marked fluctuations in these metrics reflected potential sensitivity to sample composition or limited data representation.

#### 2.5.5 Multi- and binary-class classifications

Two distinct classification strategies were used in this study. The multi-class model was trained to differentiate all classes simultaneously, whereas the binary-class model was trained to discriminate specific classes from all others independently (Chen et al., 2025; Hughes et al., 2025). Both the baseline models and the SMOTE-trained models were systematically subjected to these two configurations. Together, these alternative strategies offer complementary perspectives on the classifier’s robustness and its capacity to generalize across varying classification tasks.

## 3 Results and discussion

### 3.1 Attenuated total reflectance-fourier transform infrared analysis

**FIGURE 1:**
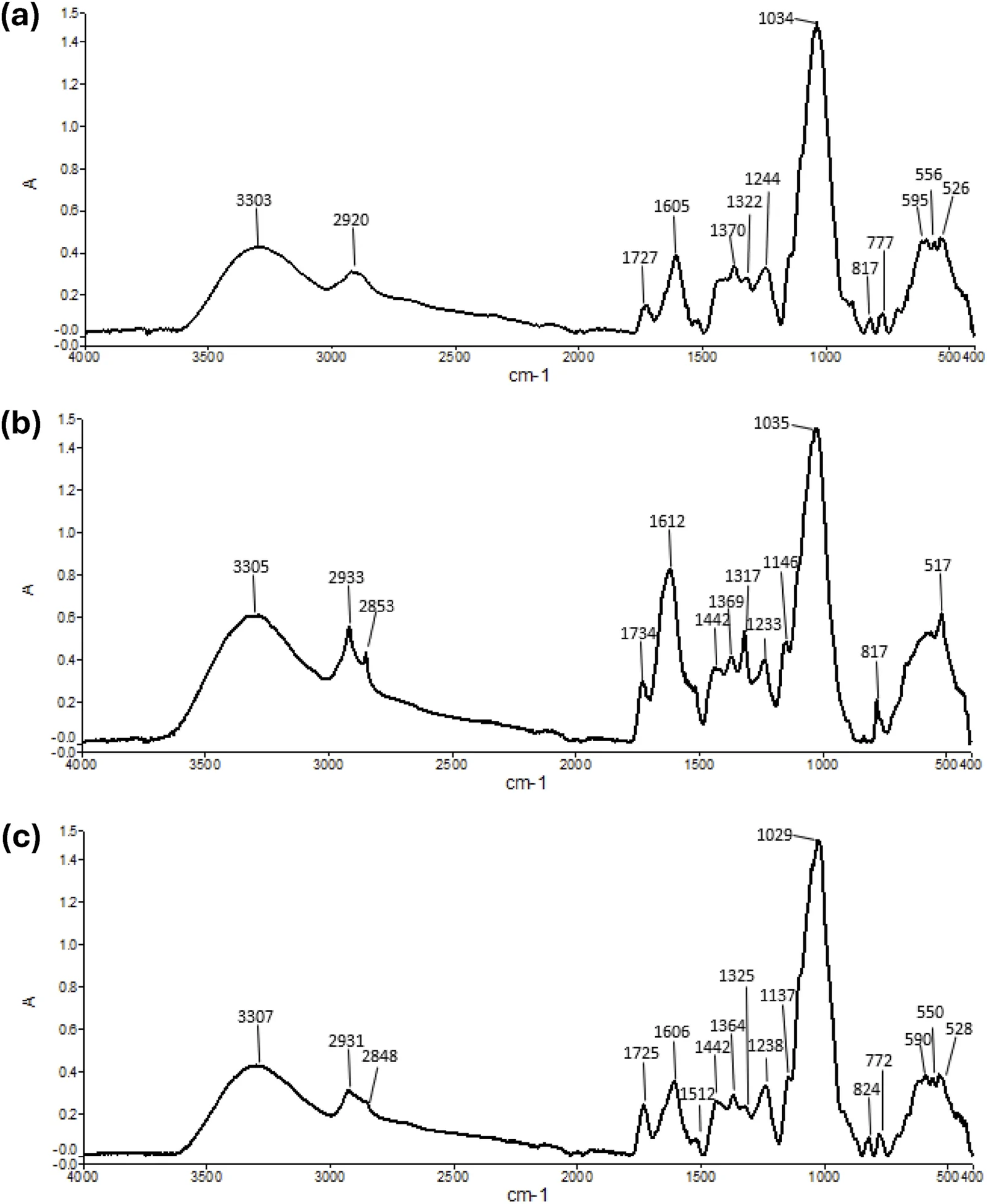
Comparison of ATR-FTIR spectra of different parts of *Camellia petelotii*: **(a)** seed, **(b)** leaf, and **(c)** flower.

The initial ATR-FTIR analysis defined the biochemical structures underlying the discriminative features of distinct parts of *C. petelotii*. As shown in Figure 1, spectral similarities were observed across distinct plant parts. The absorption peak observed in the 3,500-3,200 cm^-1^ region corresponds to O-H stretching vibrations primarily from polysaccharides or phenolic compounds (Dai et al., 2023; Lee et al., 2015). The peaks at 2,980-2,820 cm^-1^ are attributed to symmetric and asymmetric stretching of C–H in lipids. Common spectral features were further characterized by C=O stretching vibrations (approximately 1727 cm^-1^), which suggest the presence of esterified pectin or carbonyl-containing flavonoids (Han et al., 2025; Trinh, 2022). Additionally, C-H bending at 1,442 cm^-1^ and 1,370 cm^-1^, alongside C-O stretching vibrations at 1,244 cm^-1^ in esters or glycosides, were observed. The absorption peaks formed by C-O stretching vibrations associated with polysaccharides were also pronounced within the 1,200-1,000 cm^-1^ region (Fang et al., 2012; Hofko et al., 2018; Karunakaran et al., 2020; Švubová et al., 2020).

While spectral similarities were evident, the distinct spectral characteristics are crucial for discriminating among specific plant parts. The leaf of *C. petelotii* was well characterized by prominent absorption peaks at 1,612 cm^-1^ and 1,317 cm^-1^. These spectral features are tentatively attributed to the asymmetric and symmetric stretching of the COO^−^ groups of oxalate ions from calcium oxalate. Previous literature studies provide evidence that these molecular vibrations are highly characteristic of oxalate ions (Buda et al., 2018; Karimov et al., 2026; Schafer et al., 2026; Sun et al., 2011; Tew et al., 2022). The pronounced presence of calcium oxalate in the leaves is due to its physiological role as a primary site for calcium precipitation and herbivore defense. Conversely, the reproductive organs, such as seeds and flowers, prioritize storing lipids and proteins, which may explain why oxalate-associated bands are characteristic of the leaf (Graham et al., 2026). As depicted in Figure 1, the seeds and flowers shared highly similar spectral features in the lower-wavenumber region, exhibiting absorption peaks at around 817 cm^-1^, 777 cm^-1^, 595 cm^-1^, 556 cm^-1^, and 526 cm^-1^. These specific patterns have been previously associated with inorganic mineral components common to reproductive tissues (Sun et al., 2011). These distinct spectral characteristics provide discriminative information for classification. However, this wavenumber region is heavily convoluted with overlapping skeletal vibrational features from complex carbohydrates and aromatics. Therefore, definitive assignment of these specific bands to mineral constituents would require further phytochemical isolation. Moreover, the resolution constraints of ATR-FTIR may limit its ability to differentiate *C. petelotii* seeds and flowers compared to conventional KBr-FTIR. The assignments of the absorption peaks and their possible components are summarized in Table 2.

**TABLE 2:** Peak assignments in the ATR-FTIR spectra of three distinct parts of *Camellia petelotii*.

Peak			Primary assignment	Functional group
Seed	Leaf	Flower		
3,303	3,305	3,307	O-H, *v*	Various
2,920	2,933	2,931	C-H, *v*_as_	-
​	2,835	2,848	C-H, *v*_s_	-
1727	1734	1725	C=O, *v*	Ester
1,605	1,612	1,606	C-O	Oxalate/Calcium oxalate
1,322	1,317	1,325	C-O	Oxalate/Calcium oxalate
1,244	1,233	1,238	C-O, *δ*	Ester, glycosides
​	1,146	1,137	C-O	Saccharides/Glycoside
1,034	1,035	1,029	C-O	Saccharides/Glycoside
817	817	824	C-O	Saccharides/Glycoside

### 3.2 Chemometric analysis

**FIGURE 2:**
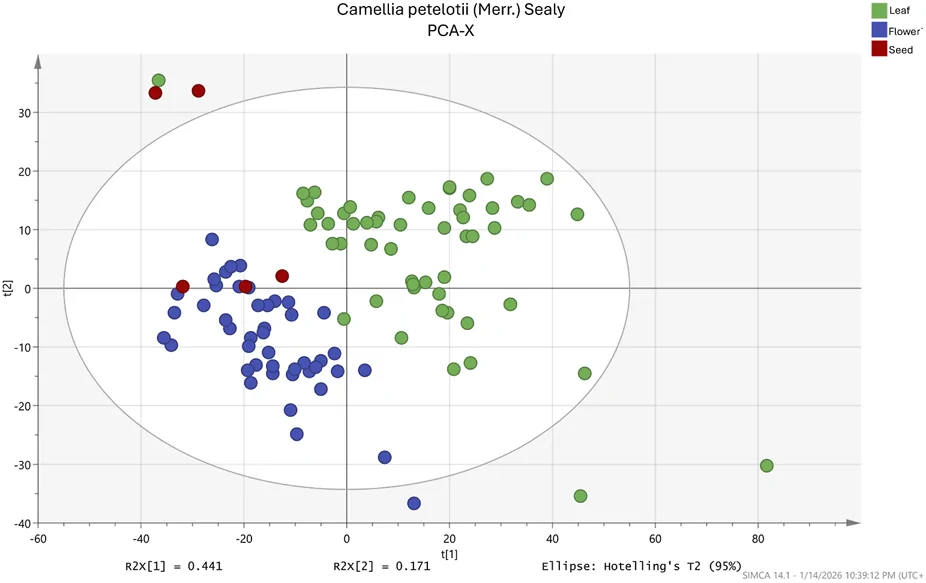
Unsupervised PCA-X score plot of different parts of *Camellia petelotii*.

**FIGURE 3:**
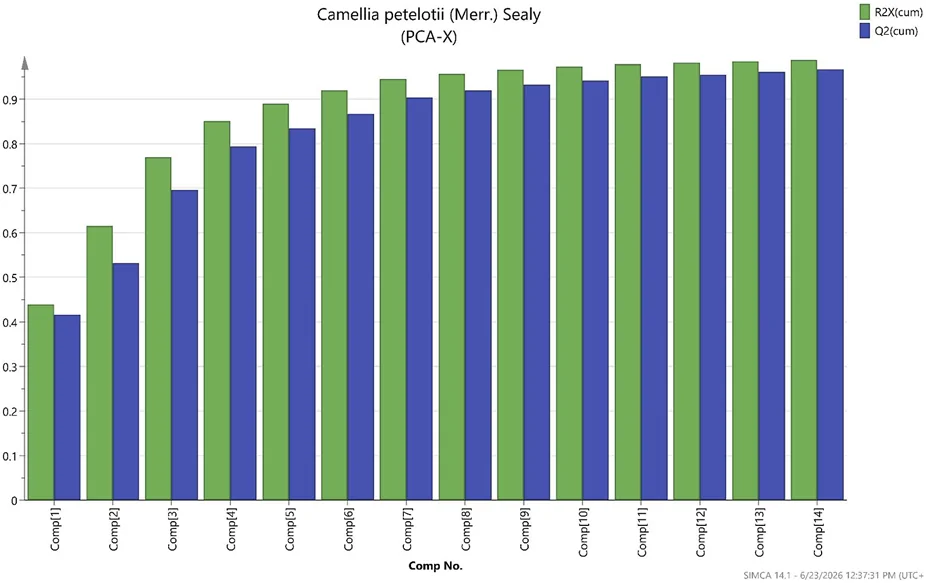
Summary of fit plot for the unsupervised PCA-X model.

Chemometric approaches were employed to demonstrate and validate the baseline distinctiveness of samples, thereby enhancing discrimination between the seeds, leaves, and flowers of *C. petelotii*. This approach applies multivariate methods for extracting actionable information from the complex spectral datasets (Brown, 2016). Principal Component Analysis (PCA) is often used as the primary tool for dimensionality reduction and visualization of clustering patterns (Jolliffe and Cadima, 2016). As depicted in Figures 2, 3, the unsupervised PCA-X model used 14 principal components to separate the samples into three distinct clusters. The model successfully captured 98.6% of the information (R^2^X = 0.986) and predicted about 96.6% of the data structure (Q^2^ = 0.966) (Table 3). Nonetheless, the PCA-class model showed differences in the intra-group homogeneity. While the Leaf and Flower classes showed high internal homogeneity (R^2^X > 0.99, Q^2^X ≥ 0.95), the Seed class showed intermediate internal coherence (R^2^X = 0.876, Q^2^ = 0.577) (Table 3). The suboptimal performance is likely due to the shortage and heterogeneity of seed samples (Rajput et al., 2023; Varoquaux, 2018). However, this variance did not affect discriminative ability, as the spectral distinctions between anatomical parts significantly outweighed intra-class differences. As shown in Table 3, the PCA-Class model achieved excellent predictive performance with an average accuracy of 98.66%, sensitivity of 99.26%, and specificity of 99.66%. This finding indicates that the spectral profiles of the leaf, flower, and seed of *C. petelotii* are distinct.

**TABLE 3:** Parameters of the PCA, PCA-class, and OPLS-DA models.

Species	R^2^X	R^2^Y	Q^2^Y	Q^2^	R^2^Y intercept	Q^2^Y intercept	RMSEE	RMSECV	RMSEP	Accuracy (%)	Sensitivity (%)	Specificity (%)
Principal component analysis (PCA-X)												
--	0.986	--	--	0.966	--	--	--	--	--	--	--	--
Principal component analysis-class (PCA-class)												
Seed	0.876	--	--	0.577	--	--	--	--	--	98.66	99.26	99.66
Flower	0.993	--	--	0.966	--	--	--	--	--			
Leaf	0.992	--	--	0.954	--	--	--	--	--			
Orthogonal partial least square-discriminant analysis (OPLS-DA)												
Seed	0.927	0.867	0.738	​	0.147	−0.278	0.124	0.181	0.091	100	100	100
Flower				​	0.129	−0.337						
Leaf				​	0.128	−0.33						

**FIGURE 4:**
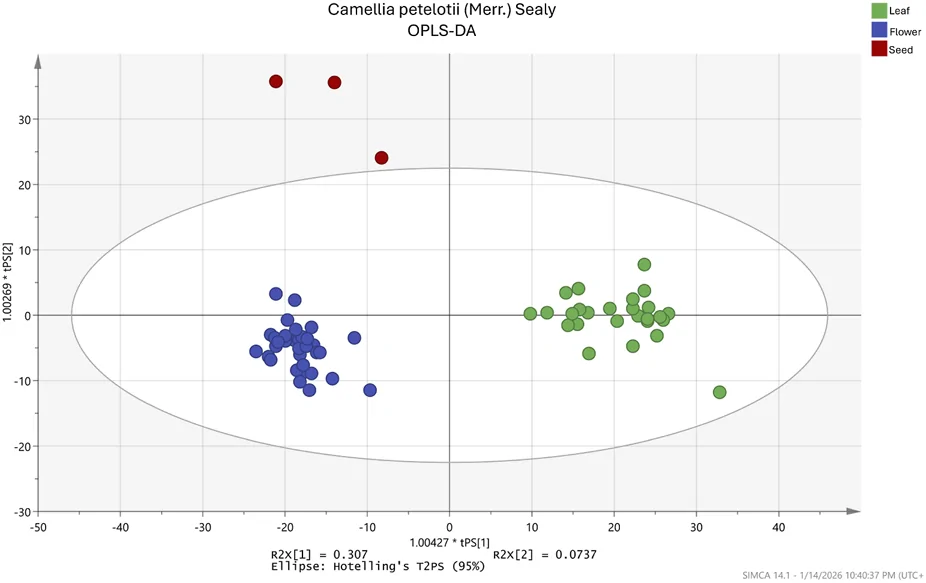
OPLS-DA predictive score plot for different parts of *Camellia petelotii.*

**FIGURE 5:**
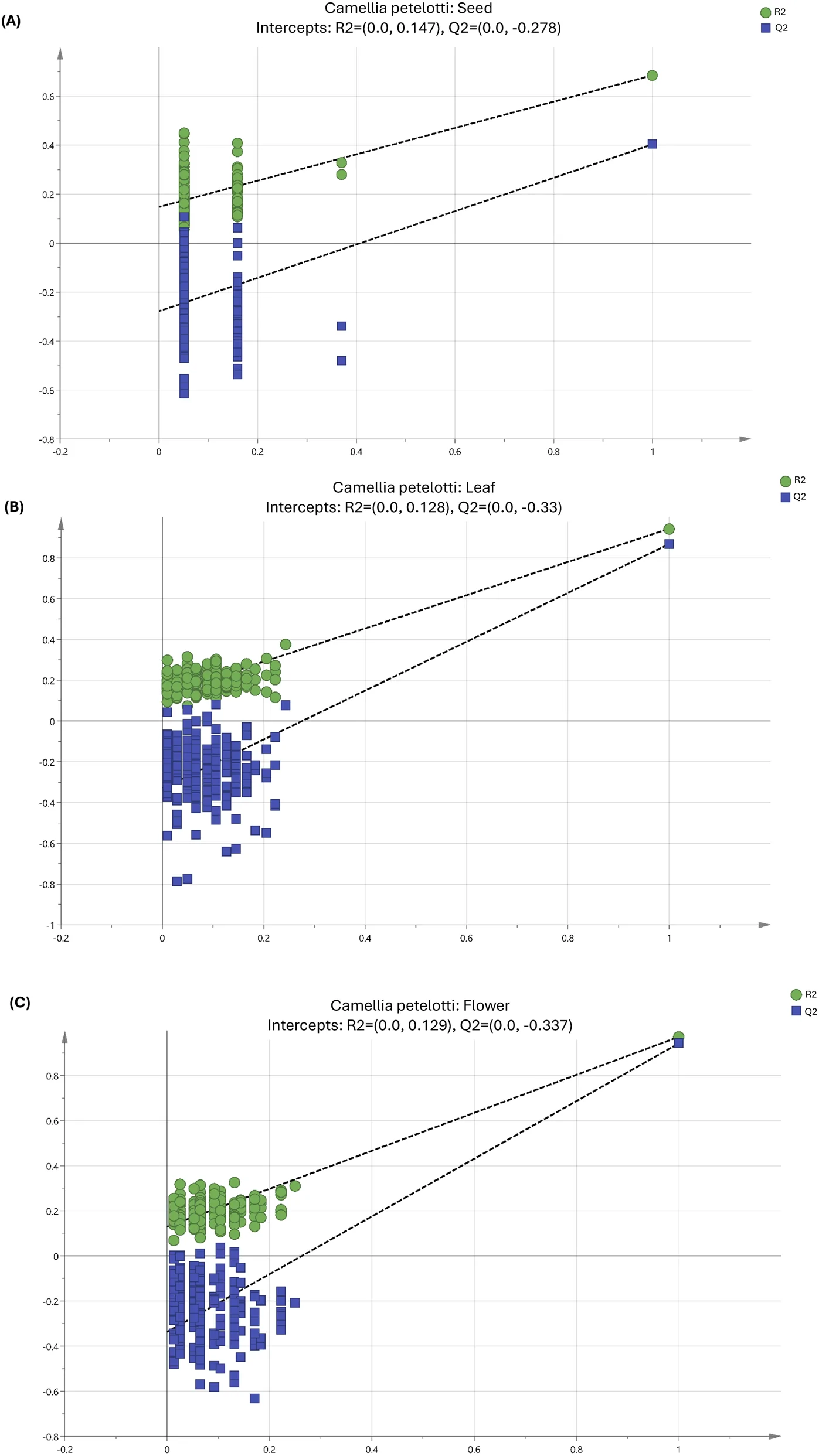
Validation of the OPLS-DA model using a 200-cycle permutation test for the **(A)** seed, **(B)** leaf, and **(C)** flower of *Camellia petelotii*.

Orthogonal Partial Least Squares Discriminant Analysis (OPLS-DA) was utilized to enhance classification. This technique separates the predictive X-variance that is relevant for class separation from the non-discriminatory variation (Chen et al., 2023; Worley and Powers, 2016). Figure 4 illustrates the strong discrimination capability of the OPLS-DA model among the leaf, flower, and seed samples. The model demonstrated high goodness of fit and predictive ability (R^2^X = 0.927, R^2^Y = 0.867, and Q^2^Y = 0.738) (Table 3). This shows that the OPLS-DA model captured over 85% of the total variance and yielded a cross-validated predictive value of 73.8%. The model’s robustness was further verified by a 200-cycle permutation test. The 200-cycle permutation test for all classes yielded R^2^Y intercepts lower than 0.15, and Q^2^Y intercepts below −0.1 (Figure 5; Table 3). The R^2^Y intercepts for seed, flower, and leaf were 0.147, 0.129, and 0.128, respectively, while their Q^2^Y intercepts were recorded at −0.278, −0.337, and −0.33, respectively. These intercept values were below the recommended thresholds (R^2^Y < 0.3; Q^2^Y < 0.05) (Table 3), indicating that the discriminative model was not overfit (Chen et al., 2023; Tew et al., 2022).

The model’s accuracy was further assessed with the parameters root mean square error of estimation (RMSEE), root mean square error of prediction (RMSEP), and root mean square error of cross-validation (RMSECV). The values for these parameters were recorded in Table 3. The low and close proximity between RMSEE (0.1406) and RMSECV (0.1733) indicate that the OPLS-DA model was robust, well-calibrated, with low overfitting (van Wyngaard et al., 2021). In addition to strong internal validation metrics, the low external predictive error (RMSEP = 0.091) indicates good generalization to the independent external test data. The low values of these parameters suggest that the OPLS-DA model shows good predictive accuracy (Tew et al., 2022). The performance metrics of accuracy, sensitivity, and specificity are predominantly used to evaluate classification precision. In the present study, the OPLS-DA model classified all classes with 100% accuracy, sensitivity, and specificity (Table 3). These metrics indicate that the chemometric model has excellent discriminative ability across all classes, even without predefined conditions (Li et al., 2018). Nonetheless, due to the small and imbalanced sample sizes across the classes, these findings were eventually used as preliminary predictive boundaries. To address this limitation and construct more robust decision boundaries against severe spectral overlap, advanced Convolutional Neural Network (CNN) models integrated with SMOTE were employed in the subsequent procedures.

### 3.3 Machine learning classification

#### 3.3.1 Training phase

**FIGURE 6:**
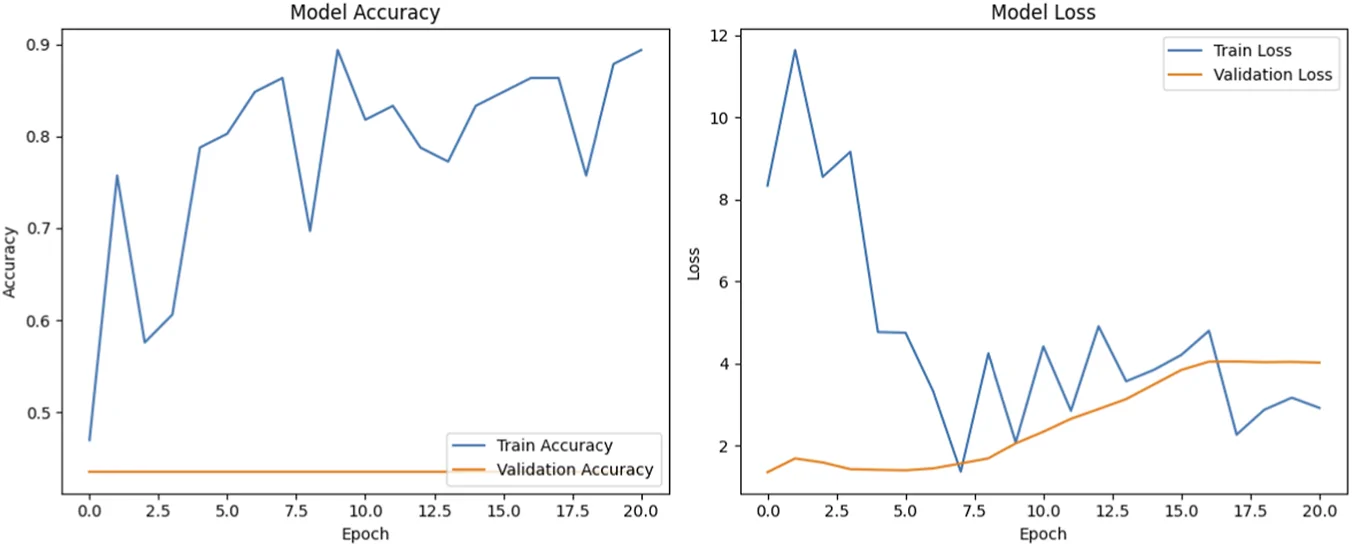
The learning curves of a representative CNN baseline model during the training process.

**FIGURE 7:**
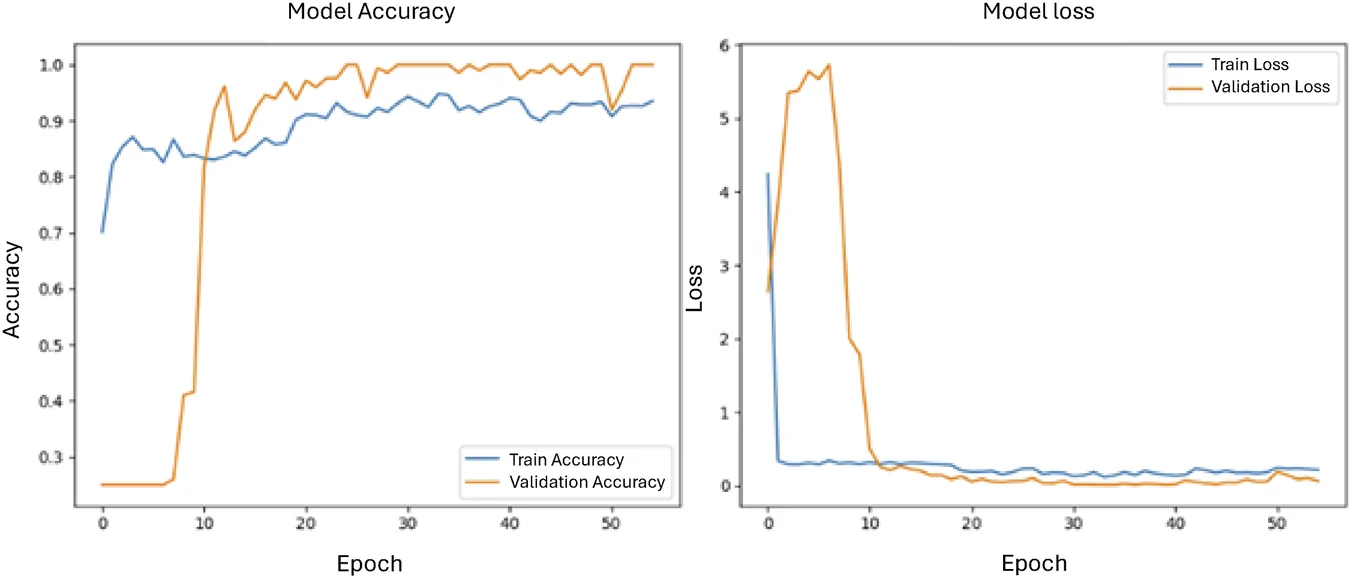
The learning curves of a representative SMOTE-trained CNN model during the training process.

Convolutional Neural Networks (CNNs) were used to address the generalization limitations in chemometrics. Figures 6, 7 present the model learning curves plotted during the training phase, which were used to assess model performance, stability, and generalization capability (Chen et al., 2025). The learning curve of the baseline models (trained on the non-augmented dataset) demonstrated pronounced instability. The validation accuracy remained low and lagged behind the training accuracy (Figure 6). Additionally, the validation loss curve failed to converge smoothly with the training loss. This phenomenon indicates that the CNN models suffered from severe overfitting and class bias with the non-augmented dataset (Buda et al., 2018; Shorten and Khoshgoftaar, 2019).

Conversely, the learning curve of the SMOTE-trained model showed that the validation accuracy rose rapidly and aligns closely with the training accuracy. Moreover, both the training and validation loss curves converged smoothly and approached zero (Figure 7). This indicates that the SMOTE-trained CNN model learned the spectral variables without signs of overfitting (Chen et al., 2025). This behavior was consistently observed across all training sessions, in both the multi- and binary-class classification experiments, indicating that the CNN models generalized well on the SMOTE-augmented training data (Johnson and Khoshgoftaar, 2019; Shorten and Khoshgoftaar, 2019). Collectively, these findings confirm that baseline models trained on non-augmented datasets failed to learn the features of minority classes with limited sample sizes.

#### 3.3.2 Evaluation phase with Repeated Random Subsampling Validation

The average F1-scores generated by multi-class and binary-class configurations were calculated and summarized in Tables 4-6. As a baseline reference, the multi-class classification CNN models trained and evaluated with the real, non-augmented dataset exhibited majority-class bias, resulting in F1-score of 0.665 for the majority Leaf class, but low F1-score for the Flower class (0.187) and near-zero score for the Mix (0.002) and Seed (0.000) classes (Table 4). Similarly, the baseline class-specific binary classification models failed to establish clear classification boundaries due to limited sample volume. This resulted in low average F1-scores of 0.228 (Flower), 0.104 (Mix), and 0.012 (Seed), but the majority Leaf class maintained a higher F1-score of 0.641 (Table 4). This scenario is attributable to the inherent behavior of convolutional networks, which favors the majority class to optimize overall accuracy. The networks tended to default to predicting the overrepresented classes, while ignoring minority classes due to insufficient sample volume and spectral variance needed to establish clear decision boundaries (Abdulsadig and Rodriguez-Villegas, 2024; Pulgar et al., 2017).

**TABLE 4:** Classification performance (F1-scores) breakdown for the multi-class and binary CNN classification models under Repeated Random Subsampling Validation.

Run	Fold	Multi-class configuration				Binary-class configuration			
		Flower	Leaf	Seed	Mix	Flower	Leaf	Seed	Mix
run_1	0	0	0.62	0	0	0.99	0.72	0	0.12
	1	0.05	0.62	0	0	0.74	0.62	0	0.12
	2	0.57	0.7	0	0	0.33	0.62	0	0
	3	0	0.62	0	0	0	0.62	0	0.12
run_2	0	0.1	0.62	0	0	0	0.62	0	0.12
	1	0.94	0.92	0	0	0	0.62	0	0
	2	0	0.62	0	0	0.35	0.62	0	0
	3	0	0.62	0	0	0	0.63	0	0
run_3	0	0.76	0.75	0	0	0.46	0.9	0	0.12
	1	0.05	0.62	0	0	0	0.62	0	0
	2	0	0.66	0	0	0.93	0.62	0	0.12
	3	0.14	0.63	0	0	0	0.62	0	0.12
run_4	0	0	0.62	0	0	0.93	0.62	0	0
	1	0	0.62	0	0	0	0.62	0	0.21
	2	0	0.62	0	0	0	0.62	0	0.12
	3	0	0.62	0	0	0	0.62	0	0.31
​	​	​	​	​	​	​	​	0	​
run_5	0	0	0.62	0	0	0	0.78	0	0.12
	1	0	0.62	0	0	0.91	0.62	0	0.13
	2	0	0.62	0	0	0	0.62	0	0.12
	3	0	0.62	0	0	0	0.62	0	0.12
run_6	0	0	0.62	0	0	0	0.65	0	0
	1	0.67	0.85	0	0.06	0	0.62	0	0.12
	2	0	0.62	0	0	0	0.62	0	0.12
	3	0.96	0.92	0	0	0.74	0.62	0	0.12
run_7	0	0.22	0.64	0	0	0	0.62	0	0.17
	1	0.78	0.77	0	0	0	0.64	0	0.13
	2	0	0.62	0	0	0	0.62	0	0.16
	3	0	0.62	0	0	0	0.62	0.33	0.12
Average		0.187	0.665	0	0.002	0.228	0.6418	0.0118	0.104
STD		0.326	0.091	0	0.011	0.366	0.062	0.062	0.073

**TABLE 5:** Classification performance (F1-scores) breakdown for the multi-class CNN classification model under repeated random subsampling validation (RRSV).

Run	Fold	SMOTE-augmented evaluation set				Non-augmented evaluation set			
		Flower	Leaf	Seed	Mix	Flower	Leaf	Seed	Mix
run_1	0	0.97	0.63	0.96	0.6	0.99	0.75	0.75	0.33
	1	0.9	0.63	0.87	0.64	0.97	0.69	0.67	0.3
	2	1	0.62	0.99	0.59	0.99	0.73	0.8	0.32
	3	0.99	0.65	0.99	0.64	0.97	0.77	0.89	0.35
run_2	0	1	0.62	0.99	0.53	1	0.79	0.89	0.3
	1	1	0.64	0.99	0.47	1	0.83	0.89	0.25
	2	1	0.63	0.99	0.52	1	0.84	0.89	0.35
	3	1	0.64	0.99	0.55	1	0.82	0.89	0.33
run_3	0	0.69	0.63	0.19	0.62	0.96	0.76	0.33	0.35
	1	0.65	0.65	0.23	0.47	0.93	0.81	0.33	0.11
	2	0.66	0.66	0.26	0.51	0.94	0.83	0.33	0.24
	3	0.68	0.66	0.25	0.48	0.95	0.87	0.33	0.29
run_4	0	0.99	0.68	0.98	0.56	1	0.88	0.89	0.43
	1	0.99	0.78	0.98	0.8	0.99	0.85	0.8	0.5
	2	0.99	0.66	0.98	0.55	0.99	0.79	0.8	0.22
	3	1	0.68	0.99	0.54	1	0.88	0.89	0.43
run_5	0	0.99	0.71	1	0.61	0.99	0.88	0.8	0.56
	1	0.99	0.69	0.99	0.66	0.99	0.88	0.8	0.56
	2	0.99	0.71	0.99	0.55	1	0.92	0.89	0.67
	3	0.99	0.63	0.9	0.71	0.99	0.83	0.8	0.48
run_6	0	0.99	0.6	0.99	0.53	0.99	0.77	0.8	0.29
	1	0.99	0.57	0.99	0.61	0.99	0.73	0.8	0.32
	2	0.99	0.52	0.94	0.25	0.99	0.77	0.67	0.21
	3	0.99	0.57	0.76	0.59	0.99	0.71	0.5	0.29
run_7	0	0.86	0.78	0.8	0.84	0.96	0.81	0.57	0.4
	1	0.96	0.76	0.94	0.77	0.99	0.83	0.75	0.42
	2	0.95	0.73	0.94	0.77	0.97	0.82	0.75	0.38
	3	0.87	0.66	0.82	0.65	0.98	0.83	0.57	0.42
Average		0.931	0.657	0.846	0.593	0.983	0.810	0.717	0.361
STD		0.115	0.060	0.263	0.119	0.019	0.057	0.192	0.120

**TABLE 6:** Classification performance (F1-scores) breakdown for the class-specific binary configuration CNN models under Repeated Random Subsampling Validation.

Run	Fold	SMOTE-augmented evaluation set				Non-augmented evaluation set			
		Flower	Leaf	Seed	Mix	Flower	Leaf	Seed	Mix
run_1	0	0.98	0.96	1	0.76	0.96	0.94	0.89	0.25
	1	0.98	0.97	1	0.74	0.96	0.95	1	0.27
	2	0.97	0.95	1	0.75	0.95	0.93	0.89	0.27
	3	0.99	0.95	0.31	0.73	0.99	0.93	0	0.28
run_2	0	1	0.95	0.99	0.86	0.98	0.93	0.8	0.33
	1	1	0.95	0.86	0.67	0.98	0.93	0.67	0.12
	2	1	0.95	0.94	0.88	1	0.93	0.67	0.38
	3	1	0.95	0.77	0.88	0.99	0.93	0.67	0.38
run_3	0	0.97	0.96	0.78	0.67	0.96	0.94	0.67	0.12
	1	0.96	0.96	0.63	0.59	0.96	0.95	0.67	0.25
	2	0.99	0.67	0	0.67	0.99	0.62	0	0.12
	3	0.96	0.96	0.59	0.59	0.95	0.95	0.67	0.26
	1	0.95	0.67	0.96	0.67	0.98	0.96	0.75	0.12
	2	0.95	0.97	0.92	0.62	0.96	0.96	0.89	0.31
	3	0.95	0.95	0.87	0.62	1	0.96	0.89	0.4
run_5	0	1	0.95	0	0.65	1	0.93	0	0.26
	1	1	0.95	0.99	0.28	1	0.93	0.8	0.17
	2	1	0.95	0	0.64	1	0.93	0	0.27
	3	1	0.95	0.99	0.36	1	0.93	0.8	0.15
run_6	0	1	0.96	1	0.17	1	0.95	1	0.11
	1	1	0.96	1	0.67	1	0.95	1	0.12
	2	1	0.96	0.77	0.67	1	0.95	0.86	0.12
	3	1	0.95	0.7	0.67	1	0.94	0.86	0.12
run_7	0	0.99	0.93	0.84	0.76	0.97	0.92	0.86	0.35
	1	0.99	0.95	0.73	0.91	0.99	0.94	0.86	0.39
	2	1	0.93	0.78	0.95	1	0.92	0.86	0.57
	3	1	0.93	0.84	0.82	1	0.92	0.86	0.37
Average		0.985	0.932	0.756	0.674	0.983	0.928	0.701	0.249
STD		0.019	0.075	0.310	0.176	0.0185	0.062	0.308	0.120

**FIGURE 8:**
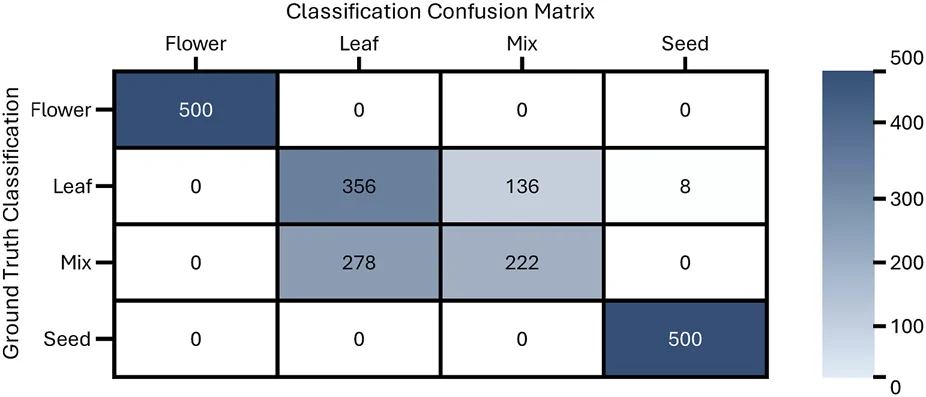
Representative confusion matrices from SMOTE-trained CNN classifiers for the multi-class configuration.

The SMOTE-trained multi-class models successfully addressed minority-class collapse (Figure 8; Table 5). When evaluated on the synthetic dataset, the multi-class model showed high classification performance for the Flower and Seed classes, with quantitative support from robust F1-scores of 0.931 and 0.846 (Table 5). Additionally, when predicting on the real, non-augmented data, these models manifested exceptional stability, achieving an average F1-score of 0.983 for the Flower class, while the Seed class demonstrated satisfactory generalization with an F1-score of 0.717 (Table 5). These results suggest that the synthetic data successfully captured the unique spectral features, enabling the Seed class to achieve robust predictive performance despite a limited number of original spectra (Chen et al., 2025; Jimenez-Carvelo et al., 2019). In contrast, inter-class confusion was observed between the Leaf and Mix classes within the synthetic prediction set. This inter-class confusion translates to lower average F1-scores of 0.593 for the Mix class and 0.657 for the Leaf class (Figure 8; Table 5). Subsequent predictions on the real evaluation dataset revealed a significant discrepancy: the Leaf class achieved a higher F1-score of 0.810, while the Mix class dropped to 0.361 (Table 5). This scenario is attributable to the intrinsic heterogeneity of the Mix class, in which composite materials contain all three plant parts, creating spectral ambiguity and affecting decision boundaries (Chen et al., 2025; Jimenez-Carvelo et al., 2019). As depicted in the confusion matrix, the CNN model systematically misclassified Mix samples as Leaf, which suggests that the leaf’s spectral features heavily dominated over the other constituent parts, driving the network to artificially inflate the pure Leaf F1-score at the direct expense of the mixture. Despite this, it remains a substantial architectural improvement over the baseline non-augmented training models, which yielded an average F1-score of 0.002 for the Mix class.

**FIGURE 9:**
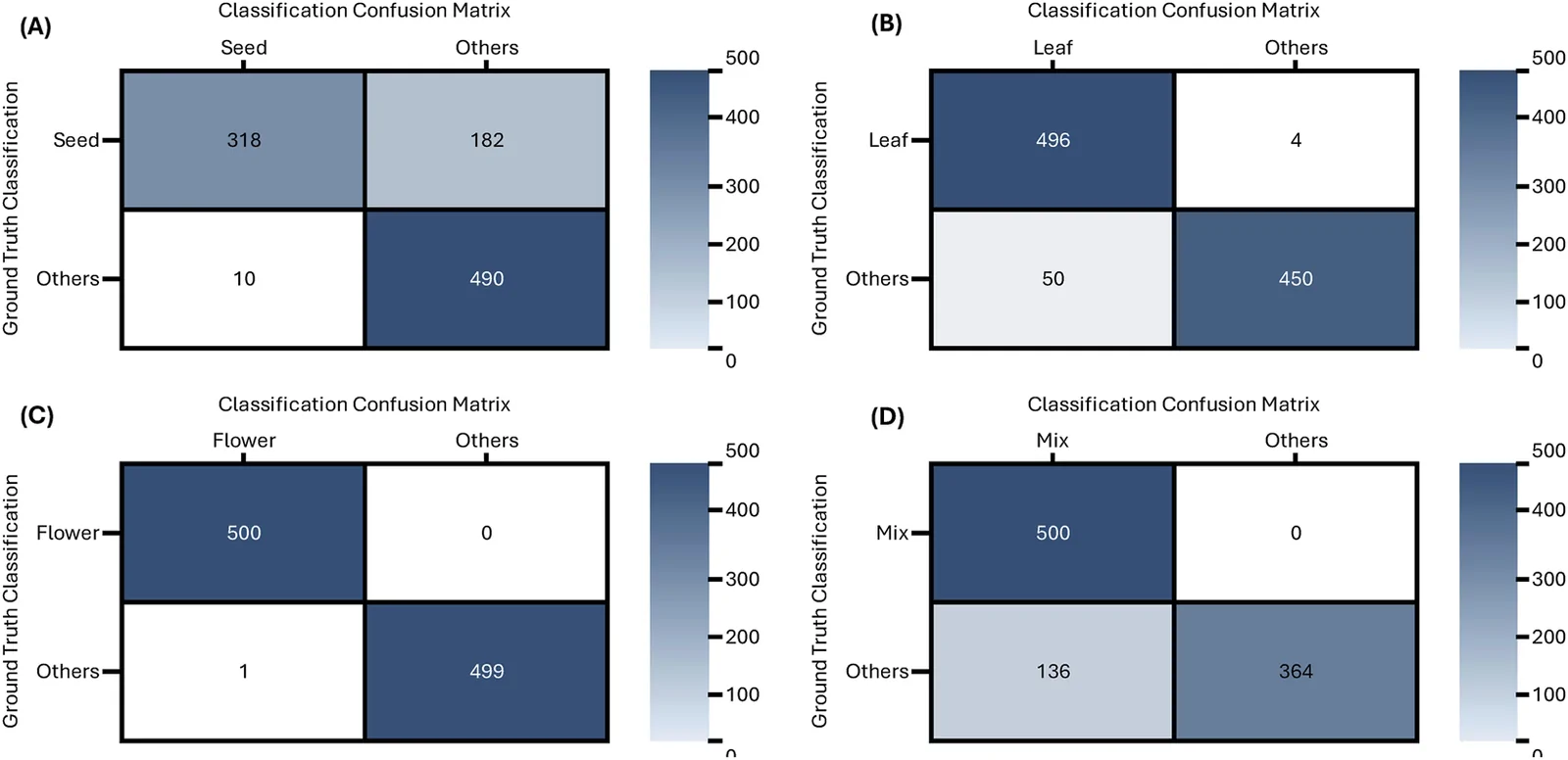
Representative confusion matrices from SMOTE-trained CNN classifiers for the binary-class configuration: **(A)** Seed, **(B)** Leaf, **(C)** Flower, and **(D)** Mix.

For class-specific binary classification, the SMOTE-trained models demonstrated a significant improvement over the non-augmented baselines and generally outperform multi-class configurations. This was evident in the Flower, Leaf, and Mix classes, where the average F1-scores rose to 0.985, 0.932, and 0.674, respectively, when predicting on synthetic data (Figure 9; Table 6). Recasting the task to binary mode simplified the decision to a single boundary, thus improving the classification performance and increasing the F1-scores (Hughes et al., 2025). However, the average F1-score for the Seed class decreased slightly to 0.756 in the binary configuration compared to multi-class models (Table 6). This condition may be attributed to class imbalance and the limited biological variance of the Seed class, with training and evaluation relying on synthetic data generated from a small pool of raw seed samples. When the model encounters the heightened spectral complexity of the “non-Seed” class, this overlapping variance compromises the model’s sensitivity toward the distinct seed matrix (Guerreiro et al., 2025; Iwendi et al., 2020).

The subsequent classification analysis of SMOTE-trained models on the non-augmented dataset showed pronounced differences in classification performance. The distinct Flower and Leaf classes remained exceptional, with F1-scores of 0.983 and 0.928, respectively (Table 6). In contrast, the F1-score of the Seed class slightly decreased to 0.701, whereas the heterogeneous Mix class experienced a severe reduction to 0.249 (Table 6). Persistent gaps remained between these heterogeneous classes and those that attained near-perfect F1-scores. This demonstrates that the main challenge stems from the class’s intrinsic heterogeneity rather than from sample scarcity. The additional synthetic data in the Mix class provided volume but not a unifying spectral signature, and did not sharpen the decision boundary against complex physical mixtures (Fernandez et al., 2018; Lohumi et al., 2015).

## 4 Conclusion

This study demonstrated the efficacy of combining ATR-FTIR spectroscopy with chemometric and machine-learning methods to differentiate parts of *C. petelotii*. The ATR-FTIR analysis defined the basic biochemical framework for discrimination by identifying unique spectral markers in each plant part. Chemometric techniques, including PCA and OPLS-DA, further interrogated the spectral information and provided effective visualization and clustering of the samples. However, the chemometric discriminative model was constrained by a limited sample size. Baseline Convolutional Neural Network (CNN) models trained on the non-augmented dataset suffered from minority-class collapse, yielding negligible F1-scores for the Mix and Seed classes. In light of this, the Synthetic Minority Oversampling Technique (SMOTE) was implemented to address this issue. Integration of SMOTE into CNN architectures successfully reduced class imbalance and improved the predictive stability of the minority Seed class in multi-class classification. Nevertheless, subsequent evaluation of SMOTE-trained models on the non-augmented dataset revealed that SMOTE provided statistical volume to optimize the decision boundaries but failed to unify spectral signatures required to classify the intrinsically heterogeneous Mix class. Conversely, recasting the classification frameworks into class-specific binary configurations enhanced the decision boundaries and increased the F1-score of the heterogeneous mixtures. Collectively, these findings suggest that classification performance is more strongly influenced by spectral specificity than by sample scarcity. The heterogeneous classes require more advanced architectural strategies to resolve the ambiguity caused by spectral overlap, to improve classification accuracy and minimize misclassification errors. Consequently, future neural networks must prioritize architectural innovations capable of resolving the profound spectral ambiguity that standard algorithmic models fail to interpret.

## Data Availability

The raw data supporting the conclusions of this article will be made available by the authors, without undue reservation.
